# Continuous glucose monitoring in people with diabetes and end-stage kidney disease—review of association studies and Evidence-Based discussion

**DOI:** 10.1007/s40620-023-01802-w

**Published:** 2023-11-21

**Authors:** Zuzanna Jakubowska, Jolanta Malyszko

**Affiliations:** Department of Nephrology, Dialysis and Internal Medicine, Warsaw, Poland

**Keywords:** Continuous glucose monitoring, Diabetes, Hemodialysis, Peritoneal dialysis, Real-time continuous glucose monitoring, Flash glucose monitoring, End-stage kidney disease

## Abstract

Diabetic nephropathy is currently the leading cause of end-stage kidney disease. The present methods of assessing diabetes control, such as glycated hemoglobin or self-monitoring of blood glucose, have limitations. Over the past decade, the field of continuous glucose monitoring has been greatly improved and expanded. This review examines the use of continuous glucose monitoring in people with end-stage kidney disease treated with hemodialysis (HD), peritoneal dialysis (PD), or kidney transplantation. We assessed the use of both real-time continuous glucose monitoring and flash glucose monitoring technology in terms of hypoglycemia detection, glycemic variability, and efficacy, defined as an improvement in clinical outcomes and diabetes control. Overall, the use of continuous glucose monitoring in individuals with end-stage kidney disease may improve glycemic control and detection of hypoglycemia. However, most of the published studies were observational with no control group. Moreover, not all studies used the same assessment parameters. There are very few studies involving subjects on peritoneal dialysis. The small number of studies with limited numbers of participants, short follow-up period, and small number of manufacturers of continuous glucose monitoring systems are limitations of the review. More studies need to be performed to obtain more reliable results.

**Graphical abstract:**

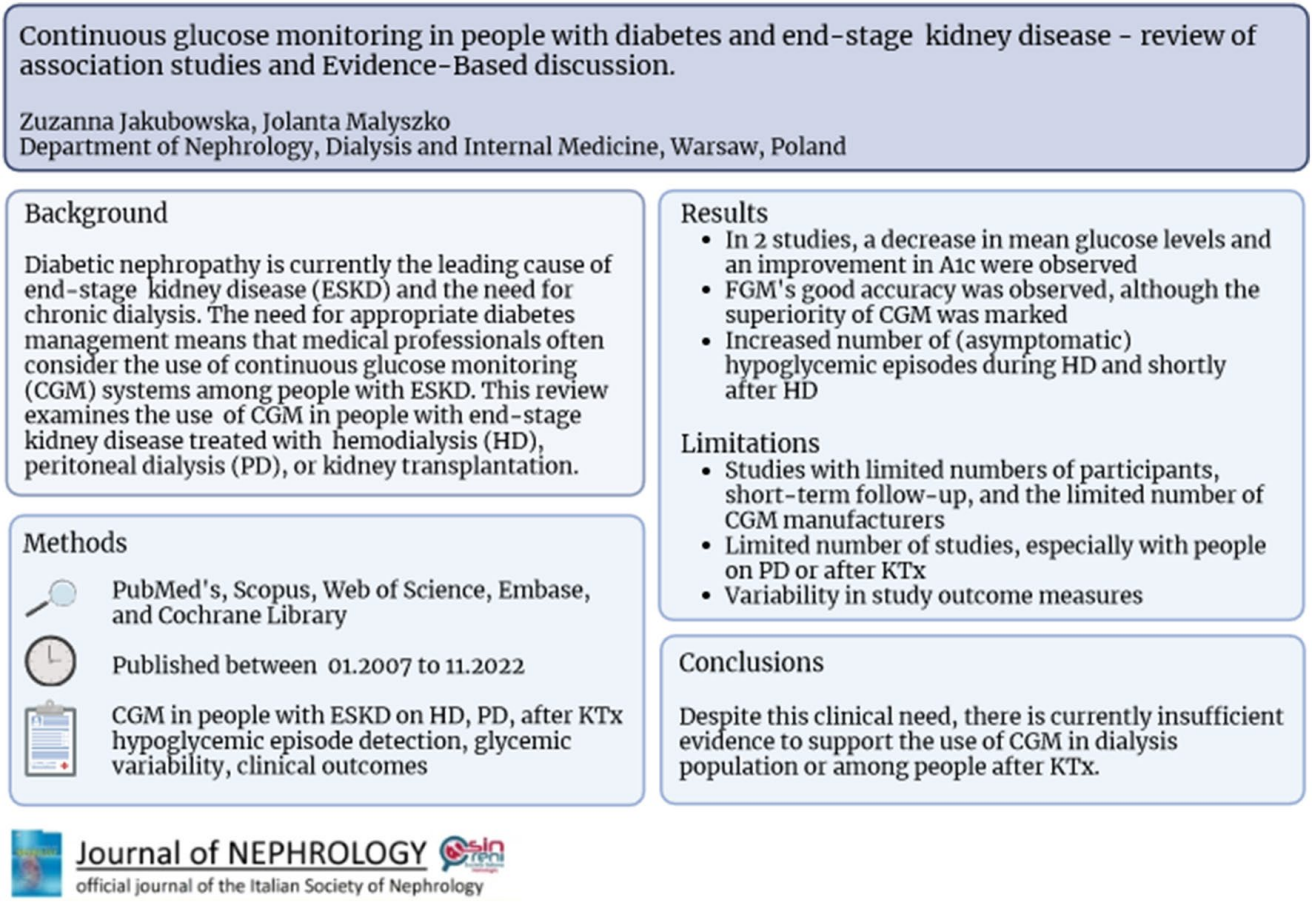

## Introduction

Diabetic nephropathy is currently the leading cause of end-stage kidney disease (ESKD) and the need for dialysis [1]. Proper glycemic control is extremely important to prevent other complications of diabetes. Moreover, in individuals with ESKD, proper glycemic control and prevention of other complications of diabetes is critical to enable kidney or simultaneous pancreas-kidney transplantation [2]. Glycated hemoglobin is one of the most common ways of monitoring glycemic control. However, especially as kidney disease progresses to ESKD, its accuracy declines [3]. Additionally, it fails to show glycemic excursions and variability. Self-monitoring of blood glucose also provides an incomplete picture of the glucose profile and may miss both hyper and hypoglycemic episodes. Self-monitoring of blood glucose is also further limited by the person's level of engagement and the burden to the person with diabetes [4]. In addition, there are also barriers to the use of self-monitoring of blood glucose reported by people with diabetes, including pain, inconvenience of testing in public places, and anxiety [5]. Given the limitations of current glucose monitoring techniques, continuous glucose monitoring may be an important tool in glycemic management in ESKD, and thus here we review its use in peritoneal dialysis (PD) and hemodialysis (HD).

Over the past decade, the technical field of continuous glucose monitoring has greatly improved and expanded [6]. Continuous glucose monitoring is a system that uses a subcutaneous sensor to measure glucose levels in the interstitial fluid. As a result, it offers continuous real-time glucose profile measurement [7]. It is also important to distinguish flash glucose monitoring, which allows continuous measurement only from regular scanning. The quality and duration of the recording depend on the involvement in the testing process [8, 9]. It has been proven that the latest models of continuous glucose monitoring and flash glucose monitoring sensors are characterized by high accuracy [10–12]. Moreover, continuous glucose monitoring is more promising in clinical practice than self-monitoring of blood glucose in terms of higher testing frequency and additional features such as assessment of glycemic trends, the use of alarms to detect abnormal glycemia (especially hypoglycemia), and the ability to track the blood glucose levels by partners, family members or healthcare professionals. Due to its widespread use in clinical practice, the use of continuous glucose monitoring improves glycated hemoglobin (A1c) and reduces glucose variability in people with type 1 diabetes and is better suited for treatment monitoring than the use of self-monitoring of blood glucose in people with type 2 diabetes [13].

Due to the limited number of publications concerning the use of continuous glucose monitoring in people with diabetes on peritoneal dialysis and after transplantation, this review mainly examines the use of continuous glucose monitoring systems in diabetics with ESKD in a chronic hemodialysis program. Single published studies on the use of continuous glucose monitoring in patients on peritoneal dialysis and after kidney transplantation are discussed in separate paragraphs. Consideration was given to hypoglycemic detection, glycemic variability and efficacy, understood as an improvement in clinical outcomes and diabetes control.

## Materials and methods

**Fig. 1:**
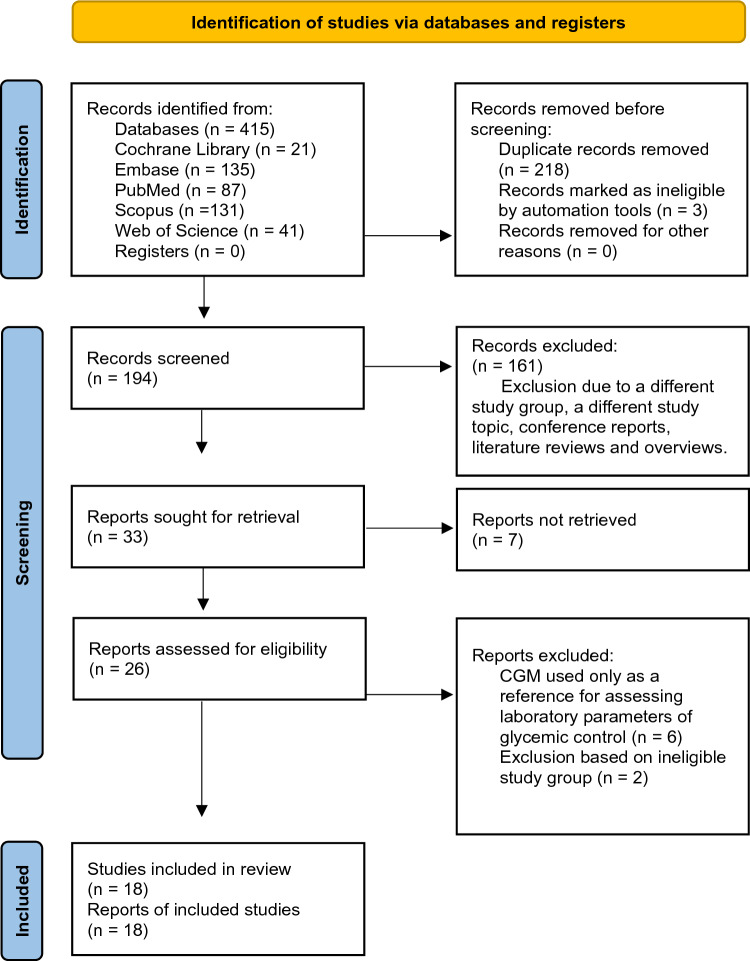
PRISMA method flow diagram of study selection. *PRISMA* Preferred Reporting Items for Systematic Reviews and Meta-Analyses

The review was conducted between 06.2022 and 11.2022. The literature review included publications on research from January 2007 to November 2022. Relevant articles were identified by two authors searching PubMed, Scopus, Web of Science, Embase, and Cochrane Library databases using advanced search and keywords: [[cgm] OR [continuous glucose monitoring]] AND [hemodialysis]]. The articles were reviewed in 3 stages by 2 researchers. We identified 415 entries, 221 of which were rejected because of duplication and by automated tools. In the second stage, 161 studies were rejected after reviewing abstracts due to an ineligible study group or an unsuitable aim of the study. Conference reports and overviews were also excluded. Finally, 18 papers were selected in the third phase. Single case reports did not qualify for the review. Figure 1 shows the schematic diagram of the study selection process. The data presented using PRISMA refer only to hemodialysis, as data for peritoneal dialysis and kidney transplant recipients are extremely limited. We found only 3 papers on continuous glucose monitoring in PD, including a report of 3 cases and another one published by the same authors with a group of 60 participants. There was only one paper on kidney transplant recipients. The materials and methods used by the authors of the qualifying studies are presented in Table 1.

**Table 1:** Summary of scientific research selected for the review with a description of the materials and methods used

	Authors	Year and type of the study	Number of participants	CGM device	Type of diabetes and treatment	Materials and methods
1.	Kazempour-Ardebili et al. [29]	2009, Prospective observational study	17	GlucoDay S	T2D /9 treated with insulin/	A 48-h CGM was performed in 17 individuals with T2D on hemodialysis to record the blood glucose profile on the dialysis day and the day after dialysis
2.	Riveline et al. [18]	2009, Prospective observational study	58 /19 HD + 39 non-HD/	MiniMed CGMS	T2D /51 treated with insulin/	In a two center study 96-h CGM was performed in 19 individuals with T2D on hemodialysis, including two days with a dialysis session and 2 days without dialysis, as well as 39 subjects not receiving HD treatment
3.	Jung et al. [27]	2010, Prospective observational study	9	MiniMed System Gold ™	T2D /7 treated with insulin/	A 144-h CGM was performed in 9 diabetics on hemodialysis to record the blood glucose profile on dialysis days and the interdialysis period
4.	Mirani et al. [24]	2010, Prospective observational study	12	GlucoDay S	T2D /12 treated with insulin/	A 48-h CGM was performed in 12 individuals with T2D on hemodialysis to record the blood glucose profile on the dialysis day and the day after dialysis
5.	Képénékian et al. [15]	2014, Prospective observational study	27	Navigator®	T2D /27 treated with insulin/	In a multi-center pilot study, a total of 54-h of CGM was performed in 27 subjects. At the beginning, a two-day glycemic recording was made, including two HD sessions and the interdialysis period, based upon which the insulin therapy was modified. After 3 months, glycemia was again recorded using the CGM method. A1c and CGM parameters were compared
6.	Chantrel et al. [16]	2014, Prospective observational study	33	Navigator®	T2D /33 treated with insulin/	A total of 48-h of CGM was performed in 33 diabetics on hemodialysis to record the glycemic profile of the dialysis day and the day after dialysis, and seven different daily periods according to meals
7.	Gai et al. [25]	2014, Prospective observational study	12	Medtronic iPRO	T2D /12 treated with insulin/	A 6-day CGM was performed in 12 diabetics on hemodialysis to assess blood glucose profiles on dialysis days and the interdialysis period
8.	Jin et al. [23]	2015, Prospective observational study	46	MiniMed MMT-7102	36 T2D /36 treated with insulin/ 10 without diabetes	A 72-h CGM was performed in 36 diabetics on hemodialysis and 10 subjects without diabetes on hemodialysis
9.	Joubert et al. [14]	2015, Prospective observational pilot study	15	Medtronic iPro2	12 T2D /10 treated with insulin/ 2 T1D	In a 12-week pilot study, 5-day CGM was performed three times in 15 diabetics on hemodialysis. Insulin therapy was adjusted on the basis of glycemic profiles obtained by CGM and SMBG methods
10.	Yajima et al. [9]	2020, Prospective observational study	13	iPro2 and FreeStyle Libre	13 T2D /8 treated with insulin/	Multi-day CGM and FGM were performed in 13 diabetics on hemodialysis
11.	Wang et al. [17]	2020, Meta-analysis	–	–	–	A meta-analysis was performed to estimate the correlation of CGM with other measures, including glycated hemoglobin A1c, glycated albumin, and mean amplitude of glucose excursions [MAGE]
12.	Toyoda, Murata, Saito et al. [11]	2021, Prospective observational study	41	FreeStyle Libre	41 T2D/without detailed information on the number treated with insulin	A 14-day FGM was performed in 41 diabetics on hemodialysis
13.	Divani et al. [22]	2021, Prospective observational study	37	Medtronic iPRO	37 treated with insulin/ without detailed information on the number with T2D and T1D	A seven-day CGM was performed in 37 diabetics on hemodialysis
14.	Hissa et al. [26]	2021, Prospective observational study	13	FreeStyle Libre	13 T2D /10 treated with insulin	A 14-day FGM was performed in 13 diabetics on hemodialysis
15.	Mambelli et al. [12]	2021, Prospective observational study	31	FreeStyle Libre	20 T2D/without detailed information on the number treated with insulin/ 11 T1D	A 14-day FGM was performed in 20 diabetics on hemodialysis and 11 individuals without diabetes on hemodialysis
16.	Hayashi et al. [21]	2021, Prospective observational study	98	System Gold Medtronic in 40 patients and Medtronic iPro2 in 58	98 T2D/65 treated with insulin, 20 treated with just oral hypoglycemic agents, 7 treated with just GLP-1A, 6 treated with diet	A two-day CGM was performed in 98 diabetics on hemodialysis to record the glycemic profile of the dialysis day and the off-dialysis day
17.	Li et al. [20]	2022, Prospective observational study	7	Medtronic iPRO	7 T2D/2 treated with insulin	A two-day CGM was performed in 7 diabetics on hemodialysis to record the glycemic profile of the dialysis day and the interdialysis period
18.	Villard et al. [19]	2022 Prospective, interventional ClinicalTrials.gov Identifier: NCT04094064	20	Dexcom G6	T2D/T1D 2 treated with insulin	A 10-day study assessed the accuracy of a factory-calibrated CGM by using venous blood glucose measurements during hemodialysis sessions and self-monitoring blood glucose at home

## Results

In two studies, a decrease in mean glucose levels and an improvement in A1c were observed as a result of long-term continuous glucose monitoring and secondary therapeutic interventions. Two studies assessed continuous glucose monitoring as a device with good accuracy due to good correlation with mean blood glucose levels. However, in three studies focusing on flash glucose monitoring, good accuracy was observed, although the superiority of continuous glucose monitoring was marked, e.g. due to accuracy deteriorating over time. Five studies found a positive correlation of A1c with average glucose levels, although it was often emphasized that these were weak correlations, unrelated to glycemic variability. In six studies, researchers observed a difference in mean glucose levels on dialysis days and non-dialysis days, with lower levels seen on dialysis days, while in two studies no differences in mean glucose levels were observed between HD and off HD days. In one of these studies, increased glycemic variability on HD days was observed. Six studies reported an increased number of hypoglycemic episodes during dialysis or during dialysis and shortly after dialysis. It was emphasized that many of these cases were asymptomatic. More details on the results are provided in Table 2.

**Table 2:** List of selected studies with a description of the results, outcomes and authors’ conclusions

	Authors	Results, outcomes and authors’ conclusions
1.	Kazempour-Ardebili et al. [29]	Mean glucose values were significantly higher the day off dialysis than the day on dialysis (12.6 ± 5.6 vs. 9.8 ± 3.8 mmol/l, *P* = 0.013). The A1C (mean ± standard deviation) was 6.9 ± 1.2% (range 5.1–9.2%), with seven subjects having A1C ≤ 6.5% Blood glucose levels were significantly lower on dialysis days than on non-dialysis days despite similar energy consumption. The risk of asymptomatic hypoglycemia was greatest within 24 h of dialysis. CGM provides much more information on glycemia compared to A1c
2.	Riveline et al. [18]	Mean glucose values were 168 ± 40 mg/dL in HD patients with T2D and 151 ± 41 mg/dL in subjects with T2D not receiving HD. A1c was 7.2% ± 1.1 in the HD T2D group and 7.7% ± 0.8 in the non-HD T2D group CGM is a validated marker of glycemic control in HD patients with diabetes with a much higher clinical value than A1c and fructosamine [in this group]
3.	Jung et al. [27]	A1c was 8.6% ± 1.2, and was correlated with mean glucose (*r* ¼ 0.780, *P* < 0.05) According to the data from CGM, HD had no effect on glucose variability. However, despite the dialysate containing glucose, HD appeared to increase the risk of hypoglycemia
4.	Mirani et al. [24]	The mean glucose value and the standard deviation (SD) of mean glucose were significantly higher in HD than in PD (186 ± 50 vs. 154 ± 25 mg/dL, *P* < 0.05 and 57 ± 26 vs. 35 ± 11 mg/dL, *P* < 0.05, respectively). Considering the 48-h recording, there was a direct correlation between the mean glucose concentration and the A1c (*r* ¼ 0.47, *P* < 0.05) Patients with diabetes treated with insulin on hemodialysis showed different profiles on dialysis days than on non-dialysis days. In particular, dialysis days were characterized by increased variability in glycemia, which may indicate an additional risk factor for cardiovascular complications. Therefore, the use of the CGM system as a method of assessing glycemic variability can improve the management of insulin therapy in these individuals
5.	Képénékian et al. [15]	The mean glucose value per CGM was 279 ± 41 at baseline and 277 ± 38 at 3 months After 3 months, A1c significantly decreased from 8.4% ± 1 to 7.6% ± 1 (*P* < 0.01) A CGM-adapted insulin regimen improves glycemic control without increasing the number of hypoglycemic episodes in hemodialysis patients with diabetes. CGM may be a useful tool for managing insulin therapy in this group, although more studies are needed
6.	Chantrel et al. [16]	The mean glucose level was 9.4 ± 2 mmol/l through all study periods, 7.5 ± 2.5 mmol/l on dialysis days and 9.4 ± 1.9 mmol/l off dialysis. A1c was 8.1 ± 1.0 mmol/mol Subjects with T2D had relatively lower glucose fluctuations compared to those with T1D. Hypoglycemic episodes during dialysis were more frequent. CGM seems to be clinically useful for the analysis of glucose profiles in this group
7.	Gai et al. [25]	At HD, the median glucose level at the start was 145 mg/dl (101–207). After the start of dialysis, all participants showed a decreasing trend of glucose with mean values falling below 100 mg/dl only after 30 min of treatment HD was associated with significant reductions in glycemia during dialysis and post-dialysis hyperglycemia. CGM devices enable better monitoring of glycemic trends in hemodialysis patients with diabetes and may improve the management of insulin therapy
8.	Jin et al. [23]	The mean glucose value was 11.12 ± 3.7 mmol/l in ESDN with SD 3.15 ± 1.12 mmol/l and 7.9 ± 2.34 mmol/l in ESKD with SD 1.34 ± 0.42 mmol/l. However, the A1c-derived mean glucose values were 9.05 ± 3.29 mmol/l in ESDN and 5.32 ± 1.16 mmol/l in ESKD Subjects with diabetes on hemodialysis had greater fluctuations in glycemia compared to idndividuals without diabetes on hemodialysis. A1c in subjects with diabetes was inaccurate, which did not reflect blood glucose levels for a long time
9.	Joubert et al. [14]	Mean glucose level was 8.3 ± 2.5 mmol/l at baseline, 8.2 ± 1.6 mmol/l at the end of the SMBG period and 7.7 ± 1.6 mmol/l at the end of the CGM period In diabetics on hemodialysis the use of CGM was associated with more therapeutic changes, and hence better diabetes control without an increased risk of hypoglycemia
10.	Yajima et al. [9]	Mean absolute relative difference of interstitial fluid glucose levels between FGM and CGM was 19.2% ± 13.8% FGM is clinically acceptable. Mean blood glucose levels were lower with FGM than with CGM
11.	Wang et al. [17]	The correlation coefficient between CGM and A1c was 0.523 [95% CI: 0.442; 604] in subjects with diabetes on dialysis and 0.592 [95% CI: 0.354; 0.757] in those with diabetes and without ESKD It has been found that in hemodialysis patients with diabetes, the use of CGM correlates well with SBMG. The correlation of CGM with A1c was similar to its correlation with glycated albumin in patients with diabetes on dialysis. Blood glucose levels were lower during dialysis compared to pre-dialysis levels
12.	Toyoda, Murata Saito et al. [11]	Sensor glucose levels and capillary glucose levels were significantly correlated (*r* = 0.858, *P* < 0.01) The accuracy of FGM in HD patients deteriorated with the days of use. Its insufficient accuracy forced the simultaneous use of FGM and SBMG
13.	Divani et al. [22]	Mean 24 h glucose was 159.2 ± 39.6 mg/dl on dialysis days and 162.4 ± 47.0 mg/dl on off-dialysis days In hemodialysis patients with diabetes, glycated albumin was more accurate than A1c. The above parameters could primarily detect hyperglycemia, but they provided limited information on the acute fluctuations in hypoglycemia and the daily variability of interstitial glucose recordings. More research is needed to fully understand whether using CGM is better than current care in improving diabetes management in hemodialysis patients
14.	Hissa et al. [26]	First-week mean glucose was 173.1 ± 78.9 mg/dl pre-dialysis and 118.58 ± 32.7 mg/dl post-dialysis. In the second week mean glucose was 154.7 ± 62.3 mg/dl pre-dialysis and 127.5 ± 56.4 mg/dl post-dialysis Mean glycemic variability in hemodialysis patients is higher than in the population without ESKD. Clinical decisions can be made on the basis of the parameters measured by CGM due to the good correlation between interstitial and capillary measurements
15.	Mambelli et al. [12]	Flash glucose monitoring and SMBG readings showed very good agreement in both T2DM and NODM (on average, 97 and 99% of readings during hemodialysis in A + B Clarke regions, respectively) FGM appears to be acceptable in monitoring glucose levels in hemodialysis patients, although the partial inaccuracy of SMBG in the control/assessment of glycemic variability requires further investigation
16.	Hayashi et al. [21]]	Mean glucose value on HD day was 150.5 ± 32.9 mg/dl and 151.0 ± 35.9 mg/dl on the off-HD day. TIR was 77.4% (1.4–100) on the HD day and 79.9% (0–100) on the off-HD day. Mean A1c was 6.4% ± 1.2 Despite the use of dialysate containing 100–150 mg/dL glucose, patients with diabetes undergoing HD experienced HD-related hypoglycemia unawareness frequently
17.	Li et al. [20]	Mean glucose value on the HD day was 6.96 ± 2.57 mmol/l and 7.86 ± 2.31 mmol/l on the off-HD day. TIR was 77.27% on the HD day and 80.39% on the off-HD day Fluctuations in blood glucose were greater on dialysis days, especially from the start of hemodialysis up to 2 h post-dialysis, than on non-dialysis days. Hypoglycemia occurred more frequently on dialysis days than on non-dialysis days
18.	Villard et al. [19]	Time in range was 38.5% (interquartile range 29.3–57.9), with 28.7% (7.8–40.6) of the time > 250 mg/dL CGM appears to be a fairly accurate and clinically relevant option for use in practice by hemodialysis users and healthcare professionals to improve diabetes management

## Discussion

### Continuous glucose monitoring and improvement in diabetes management

Proper diabetes control is essential to prevent or delay the chronic complications of diabetes, and to maintain a higher quality of life and longer life expectancy. In addition to a balanced diet and physical activity, the most important factors are the proper assessment of glycemia and the correct supply of insulin. One of the methods of glycemic assessment could be continuous glucose monitoring. In a study by Joubert et al. the use of continuous glucose monitoring was associated with more therapeutic changes, and hence better diabetes control [14]. However, it must be emphasized that this was an observational pilot study involving only 15 people on dialysis from one center, without a control group. Based on this study, it cannot be unequivocally concluded that continuous glucose monitoring improves glycemic control in individuals with ESKD. In a study by Képénékian et al., where continuous glucose monitoring was used with a 3-month follow-up, the A1c and continuous glucose monitoring glucose levels decreased without an increase in the number of severe hypoglycemic episodes [15]. However, this improvement was noted on the basis of a significantly reduced A1c level, which is not an ideal parameter for glycemic control in people on hemodialysis. Even if no changes in hemoglobin were observed, the risk of misjudgment is high. Chantrel et al. also emphasized that self-monitoring blood glucose measurements and A1c levels are of limited value in hemodialysis patients with diabetes [16]. To optimize glycemic control in these individuals, continuous glucose monitoring may be worth considering [17]. A study by Riveline et al. highlighted that A1c and fructosamine, although good markers of glycemic control in non-hemodialysis individuals with diabetes, are of limited value in diabetic subjects on HD and that it is worth considering the use of continuous glucose monitoring in this group [18]. The use of the data may also have influenced the outcome of diabetes control. In some studies, previously trained individuals manually self-adjusted insulin therapy, in the study by Joubert et al. there was remote therapeutic counseling, in other studies therapeutic decisions were made by medical professionals [14]. One important aspect, not often addressed in the above sources, is the issue of confounding factors. Factors that might interfere with the use of continuous glucose monitoring in patients with ESKD include amblyopia secondary to diabetic retinopathy. Self-application of the continuous glucose monitoring system in people with advanced vision problems is more difficult than self-monitoring of blood glucose. In addition, not all receivers have voice control, which excludes people with visual impairment from everyday use of continuous glucose monitoring. Another issue is the difficult application of continuous glucose monitoring in people with malnutrition and diminished subcutaneous adipose tissue. In these people, the available skin surface suitable for proper application is significantly reduced. Frequent changes of application site are essential to avoid local side effects. Moreover, there is little data available on the accuracy of measurements in adults with reduced subcutaneous fat. Another issue is that patients on chronic dialysis often develop disorders of calcium-phosphorus metabolism. A common symptom in individuals with hyperphosphatemia is pruritus, which may be further exacerbated by the device and patches applied to the skin, or frequent allergic reactions to the adhesive components of the continuous glucose monitoring patch. In addition, a foreign body always carries a higher risk of infectious complications in immunocompromised subjects, such as those with ESKD, who are more frequently exposed to infections, are in large groups in dialysis centers, and have frequent contact with the hospital environment. With regard to the peritoneal dialysis patient group, icodextrin peritoneal dialysis solutions may affect the accuracy of continuous glucose monitoring results. To conclusively assess the potential improvement in diabetes control, a broader study including a group with the above complications of diabetes and dialysis should be conducted.

### Continuous glucose monitoring and glycemic variability on hemodialysis days and non-hemodialysis days

High variability in blood glucose levels beyond the range of euglycemia has been identified as an important risk factor for microvascular and macrovascular complications, hypoglycemia, and mortality [13]. Therefore, it seems important to assess the variability of glycemia in hemodialysis patients, especially in the context of the differences between dialysis days and non-dialysis days. One helpful parameter for assessing the differences between hemodialysis days and interdialytic period could be the time in range. Unfortunately, researchers only used the time in range parameter in four studies. In a study by Villard et al. the median time in range (70–180 mg/dL) was 38.5% (interquartile range 29.3–57.9), with 28.7% (7.8–40.6) of the time > 250 mg/dL [19]. However, in the study by Li et al. on the day of hemodialysis the time in range was 77.27%, and on the day without hemodialysis it was 80.39% [20]. Similarly, in a study by Hayashi et al. average time in range was 78.7% and time below range was significantly higher on the HD day than on the non-HD day [21]. In 2021, Divani et al. observed that time in range value was 62.2% ± 22.3 on HD days and 65.2% ± 27.5 on off-HD days [22]. Other publications mainly used mean glucose and standard deviation. In a study by Jin et al., which compared a group of people with and without diabetes, it was shown that in the former group the variability of blood glucose was much greater, and that a basic glycemic control parameter, such as A1c, did not show this variability [23]. In a study by Mirani et al. 12 hemodialysis patients with diabetes treated with insulin showed different glycemic profiles on dialysis and non-dialysis days [24]. Similarly, the study by Gai et al. demonstrated significant variability in blood glucose levels on the day of dialysis, and recommended closer monitoring at the end of the dialysis session and immediately after dialysis, as well as possible adjustments to insulin therapy at that time [25]. According to Li et al. fluctuations in glycemia were higher on dialysis days, especially from the start of hemodialysis up to 2 h after hemodialysis, than on non-dialysis days [20]. A study by Hissa et al. reported that the variability of glycemia in hemodialysis patients is higher than in the non-ESKD population [26]. Because of the good correlation between interstitial and capillary measurements, it was recommended that clinical decisions be made on the basis of values measured by continuous glucose monitoring. In contrast, hemodialysis had no effect on glycemic variability in the study of Jung et al. involving nine participants, according to the continuous glucose monitoring data. [27] Despite some differences in the results of these studies, it appears that continuous glucose monitoring may be a useful method for assessing glycemic variability and managing therapy in individuals on hemodialysis.

### Continuous glucose monitoring and detection of hypoglycemia

Hypoglycemia is when the blood glucose level falls below 3.9 mmol/l (< 70 mg/dl). [28] It includes mild hypoglycemia, which the person can control by taking carbohydrates, and severe hypoglycemia, which requires the help of another person to stop it. Severe hypoglycemia can lead to permanent neurological complications and death. Therefore, it is very important to determine whether HD increases the risk of hypoglycemia and whether the use of continuous glucose monitoring can be a beneficial method for more frequent detection of possible glycemic episodes in diabetic patients on hemodialysis. Data from the study by Joubert et al. indicate that the use of continuous glucose monitoring provides more comprehensive insight into the glycemic profile and better adjustment associated with a grater number of therapeutic changes without increasing the risk of hypoglycemia [14]. According to Kazempour-Ardebili et al. the risk of asymptomatic hypoglycemia was highest within 24 h of dialysis regardless of similar caloric intake [29]. Therefore, it was emphasized that healthcare professionals who provide health services to individuals on hemodialysis need to be aware of this phenomenon and should consider increased monitoring of blood glucose levels following hemodialysis sessions. Similar conclusions were drawn in the study by Gai et al [25]. According to Chantrel et al. hypoglycemic episodes occurred more frequently during the dialysis session, therefore the use of continuous glucose monitoring was also recommended [16]. A study by Jung et al. also showed that HD seemed to increase the risk of hypoglycemia [27]. Multiple hypoglycemic episodes, often asymptomatic, were detected in 80% of individuals on the day of dialysis, especially within the first 12 h after dialysis initiation. The conclusions from the study by Li et al. were similar, but each episode was shown to be asymptomatic [20]. Mori et al. [30] described the case of a 68-year-old Japanese man who had been undergoing HD for 18 years and had diabetes for 41 years, treated with a premixed insulin analog. Continuous glucose monitoring showed relatively flat 24-h blood glucose levels on non-HD days, while an acute drop in blood glucose level occurred during the HD session, and a subsequent severe blood glucose level elevation was observed after the HD. They stated that such phenomena might be comparable to HD-induced hypoglycemia and HD-associated hyperglycemia. They also added that continuous glucose monitoring was a powerful tool to visualize blood glucose fluctuations caused by various medications. These findings described by Mori et al. [30] were further supported by Matoba et al. [31] who studied simultaneous flash glucose monitoring (FreeStyle LibrePro), continuous glucose monitoring (iPro2) and self-monitoring blood glucose in 13 hemodialysis patients with type 2 diabetes mellitus. They found that the accuracy of flash glucose monitoring compared with self-monitoring blood glucose was worse than that of continuous glucose monitoring. In a study by Jin et al. in CGM more episodes of asymptomatic hypoglycemia were detected, emphasizing that other parameters such as A1c or glycated albumin did not show the presence of the above episodes. [23] This increased risk of hypoglycemia in most studies, even when performed on a small number of participants, prompts efforts to increase safety after hemodialysis sessions.   

### Continuous glucose monitoring and glycemic variability in patients on peritoneal dialysis

Achieving adequate glycemic control in diabetic patients on peritoneal dialysis is challenging. Similarly to those on HD, conventional assessment of glycemia using A1c is difficult because of renal anemia or carbonylation of hemoglobin, and significant glucose excursions may be masked [32]. In addition, there is concern that conventional dialysate solutions utilizing supraphysiological concentrations of glucose as their osmotic agent may contribute to this increased cardiovascular risk [32]. Cardiovascular risk depends upon membrane characteristics and dwell time in people on PD. The systemic consequences of glucose absorption are not well understood. Peritoneal dialysis is a continuous therapy, people are exposed to variable concentrations of glucose during their prescription and are never truly ‘fasted’ unless PD is withheld. The best choice of a biomarker for measuring the additional metabolic burden in these people remains uncertain.

Data on continuous glucose monitoring in the PD population is very limited. The report of Oei et al. [33] described three patients with diabetes on peritoneal dialysis with similar A1c levels, but with very different glucose profiles as shown by continuous glucose monitoring. The authors suggested that intermittent continuous glucose monitoring might allow safer management of glycemia in patients on PD because none of them were aware of hypoglycemia during the periods of low glucose recorded on continuous glucose monitoring. The same group extended their observation and retrospectively analyzed 60 PD patients with diabetes treated with insulin [33]. They found only a weak correlation between A1c levels and mean glucose levels measured by continuous glucose monitoring. They emphasize that A1c level is an inadequate indicator of glycemic control in diabetics on PD and suggested that the continuous glucose monitoring technology should be more widely adopted. The most recent study by Williams et al. [34] included 15 patients without diabetes on PD and 16 patients with stage 5 chronic kidney disease as controls undergoing 72 h of continuous glucose monitoring. The researchers found that automated peritoneal dialysis was associated with significantly higher nocturnal blood glucose than continuous ambulatory peritoneal dialysis. In addition, the significant drop in nocturnal blood glucose compared with the daily average observed in both continuous ambulatory peritoneal dialysis participants and control subjects was not observed in automated peritoneal dialysis patients.

### Continuous glucose monitoring in kidney allograft recipients

In the only published study on 24 kidney allograft recipients, Jin et al. [35] investigated the glucose profiles and assessed the degree of hyperglycemic excursion during the early period after surgery. They found that hyperglycemia over 126 mg/dL (fasting) or 200 g/dL (postprandial) occurred in 42.1% (8/19) of kidney transplant recipients during this early period after transplantation, except for patients with preexisting diabetes (5 patients). They suggested performing more studies involving continuous glucose monitoring follow-up at regular intervals based on the time since transplantation.

## Conclusions

The above literature review provided some important information on the use of continuous glucose monitoring in diabetic patients on hemodialysis, especially those with hypoglycemia. This review has some limitations due to the limitations of the conducted studies, which include the limited number of participants, short-term follow-up, and the limited number of continuous glucose monitoring manufacturers. Most studies did not have a control group and were strictly observational. Moreover, not all studies used the same assessment parameters. Most of the studies also did not refer to the Diabetes Care recommendations in their assessment parameters [35], which would help to clarify the importance of continuous glucose monitoring in the treatment of diabetic patients on hemodialysis. In addition, data from studies on the use of continuous glucose monitoring in patients with diabetes and ESKD treated with peritoneal dialysis, and kidney transplant recipients were collected.

After the selection of articles was completed, during the work on this review, many significant articles were published on the accuracy, usefulness, and safety of continuous glucose monitoring in diabetics on hemodialysis and peritoneal dialysis. A study by Horne et al. conducted on a group of 69 hemodialyzed patients with the use of flash glucose monitoring analysis showed 97.9% of glucose values in an acceptable range of agreement. Measurements were especially accurate on non-dialysis days. These findings show that flash glucose monitoring can be as accurate as self-monitoring of blood glucose or laboratory serum glucose [37]. In another study, researchers compared the accuracy of Dexcom G6 continuous glucose monitoring and Freestyle Libre 1 flash glucose monitoring using the Yellow Springs Instrument (YSI) method. Analyses showed that the mean absolute relative difference for continuous glucose monitoring was 22.7% and 11.3% for flash glucose monitoring. As a result, flash glucose monitoring was found to be a very reliable tool in everyday clinical use, but more research is needed on the safety and accuracy of the Dexcom G6 in this group [38]. In a study by Jack K.C. et al. on 30 patients on PD, continuous glucose monitoring Medtronic Guardian Sensor 3 was shown to be accurate, with an overall mean absolute relative difference of 10.4% using YSI as the gold standard reference for the assessment of continuous glucose monitoring accuracy [39]. This shows that continuous glucose monitoring seems to be accurate in measuring glucose levels in PD patients. In the study by Shah et al., the focus was on glucose levels during HD and post-HD. Targeting pre-HD glucose levels below 180 mg/dl has been shown to be appropriate to prevent large fluctuations during and after HD [40]. In a study by Bomholt et al. on a small group of HD patients, albeit over a longer follow-up period, no significant differences were found between the average glycemia levels between dialysis and non-dialysis days [41]. In another review article, Bomholt et al. emphasized that continuous glucose monitoring does not share the flaws of HbA1c in HD patients, and that the recommended minimum of 50% time spent in the target range (3.9–10.0 mmol/L) and less than 1% below range (< 3.9 mmol/L) seems to be a better target for the management of diabetes in HD patients [42]. In another review article, Williams et al. emphasized that further studies on continuous glucose monitoring in dialysis patients are necessary because in other populations it is currently the gold standard of glycemic assessment and it is required to determine whether this technology can improve clinical outcomes in HD patients [43].

Despite this clinical need, there is currently insufficient evidence to support the use of continuous glucose monitoring in the dialysis population. We recommend that larger multi-center trials be conducted in a larger group including different types of diabetes, and that the latest continuous glucose monitoring models should be used for longer than the usual 48–72 h. It would be reasonable to include people with disabilities characteristic of ESKD, such as amblyopia secondary to diabetic retinopathy, and people with lower body weight and other confounding factors mentioned above. In addition, we recommend including a quality of life and cost-effectiveness analysis.

The most recent international consensus statement presents recommendations optimizing continuous glucose monitoring-derived glucose data collection in clinical studies, including the specific glucose metrics that should be evaluated. [36] These recommendations have also been endorsed by the American Association of Clinical Endocrinologists, the American Diabetes Association, the Association of Diabetes Care and Education Specialists, Diabetes India, the European Association for the Study of Diabetes, the International Society for Pediatric and Adolescent Diabetes, the Japanese Diabetes Society, and the Juvenile Diabetes Research Foundation [44]. Thanks to a standardized approach to continuous glucose monitoring data collection and reporting in clinical trials, the ability to interpret continuous glucose monitoring data will be enhanced, providing useful information for therapeutic decision making, in particular related to hypoglycemic episodes, postprandial hyperglycemia and glucose variability.

## Data Availability

The data underlying this article are available in the article.
